# Drug-Drug Interaction of Warfarin and Doxycycline Leading to a Large Rectus Sheath Hematoma

**DOI:** 10.7759/cureus.81312

**Published:** 2025-03-27

**Authors:** Shadrack Ansong, Jagroop Doad, Emmanuelar O Igweonu-Nwakile, Chika Okafor

**Affiliations:** 1 Internal Medicine, Cape Fear Valley Medical Center, Fayetteville, USA; 2 Internal Medicine, Campbell University School of Osteopathic Medicine, Lillington, USA

**Keywords:** doxycycline, drug-interaction, endovascular embolisation, rectus sheath hematoma, supra-therapeutic inr recurrent valve thrombosis, warfarin therapy

## Abstract

In recent years, there has been a significant reduction in the use of warfarin, as many patients have transitioned to direct oral anticoagulants (DOACs) for the management of atrial fibrillation. However, a considerable number of patients continue to rely on warfarin due to financial limitations and the specific requirement for its use in individuals with mechanical heart valves, given the insufficient data on the effectiveness of DOACs in these scenarios. While warfarin is recognized for its high efficacy, it possesses a narrow therapeutic window, necessitating careful monitoring to avoid excessive bleeding. Consequently, regular assessments of the international normalized ratio (INR) are essential to ensure that anticoagulation remains within the therapeutic range, guiding appropriate dosage adjustments. This report details a case involving a 69-year-old male with a history of atrial fibrillation who was on warfarin therapy and subsequently developed a significant rectus sheath and extraperitoneal hematoma after receiving doxycycline for pneumonia. His INR was recorded at over 8 (facility laboratory limit highest at the time of this case). He was initially managed with vitamin K and prothrombin complex concentrate, followed by interventional radiology consultation for embolization of the right inferior epigastric artery due to the presence of an expanding rectus sheath hematoma observed on CT imaging. Doxycycline may potentiate the anticoagulant effects of warfarin by competing for albumin binding, which may elevate the risk of severe bleeding complications. Therefore, it is imperative to exercise caution when prescribing doxycycline to patients undergoing warfarin therapy.

## Introduction

Warfarin is an oral anticoagulant extensively utilized to prevent the formation of blood clots in various medical scenarios. Despite ongoing initiatives by healthcare professionals to shift patients from warfarin to direct oral anticoagulants (DOACs), largely due to the latter's more favorable side-effect profile, warfarin continues to be one of the most frequently prescribed medications in the United States [1,2]. In 2021, more than 11 million prescriptions for warfarin were issued [1]. While warfarin offers certain advantages, such as lower cost, it poses risks of minor to potentially life-threatening bleeding if not maintained within its narrow therapeutic range. Although it is recognized for its high efficacy, warfarin requires careful monitoring due to its limited therapeutic window to achieve the desired anticoagulation effect without increasing the risk of excessive bleeding [3]. Consequently, regular monitoring of the international normalized ratio (INR) is essential to assess the drug's effectiveness and to guide appropriate dosing, whether daily, weekly, or monthly.

The annual rate of bleeding incidents associated with warfarin is reported to be 7.6 per 100 patient-years [3,4]. Analyses indicate that cutaneous bleeding and macroscopic hematuria are the most prevalent major and minor bleeding events among warfarin patients presenting to emergency departments [2,5]. Notably, abdominal hematomas appear to be rare, with only a few documented cases in the literature [5]. Although there are specific guidelines for managing hemorrhagic complications linked to warfarin, most recommendations advocate for the immediate reversal of anticoagulation, typically achieved through the administration of vitamin K and factor replacement using prothrombin complex concentrate or fresh frozen plasma [3,4,5]. However, a specific protocol for rectus sheath hematoma has not been formulated. This report details a case involving a rectus sheath hematoma that expanded into the space of Retzius in a patient compliant with warfarin therapy, following the completion of a doxycycline course for pneumonia.

## Case presentation

A 69-year-old male with a medical history notable for atrial fibrillation managed with warfarin for the past fifteen years, obstructive sleep apnea, type 2 diabetes mellitus, chronic kidney disease stage 3B, and a history of esophageal cancer currently in remission, presented to his primary care physician due to one day of right flank bruising, abdominal discomfort, and groin pain. The patient had been on 5 mg of warfarin every Monday, Wednesday, and Friday and 3 mg Tuesday, Thursday, Saturday, and Sunday, with his last INR check being three days before he was started on antibiotics, as stated below, with the INR being 2; he has been averaging between 2 and 2.6 for his last four INR checks. Additional relevant history included a recent pneumonia diagnosis within the last two weeks, for which he was undergoing an outpatient treatment regimen with doxycycline. The patient was diagnosed with community-acquired pneumonia and was started on doxycycline 100 mg twice daily for seven days. On the fourth day of the start of the doxycycline, the patient called and reported abdominal discomfort and bruising to his primary care physician. The primary care physician (PCP) ordered a contrast-enhanced computed tomography (CT) scan of the abdomen and pelvis and informed the patient that he would be contacted with the results. After the CT scan revealed a rectus sheath hematoma, the PCP promptly reached out to the patient, advising him to visit the emergency department. The patient subsequently went to the nearest emergency department.

**Figure 1:**
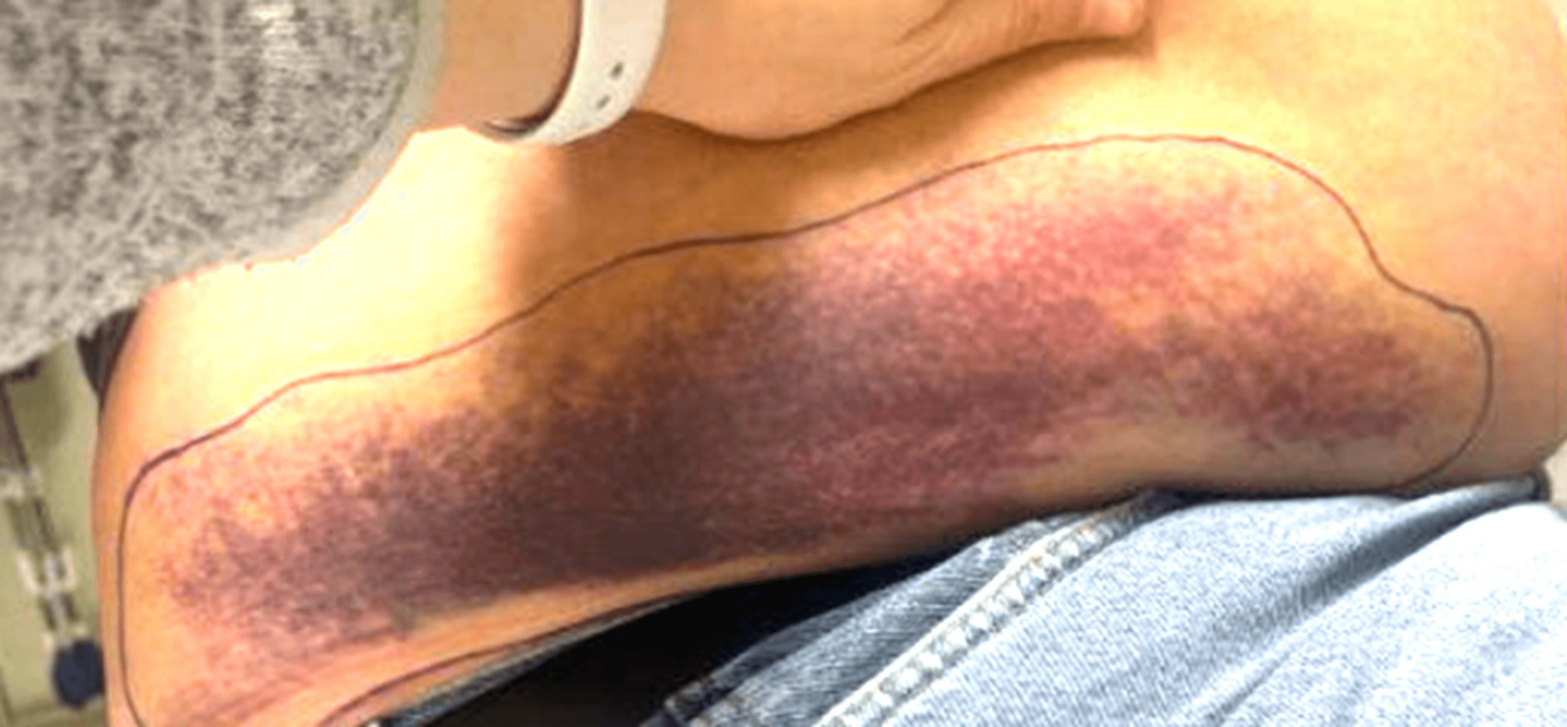
Large ecchymosis noted in the right flank region.

Upon his arrival, the patient was hemodynamically stable and exhibited no signs of acute distress. A physical examination revealed a distended abdomen and ecchymosis in the right flank (Figure 1).

**Figure 2:**
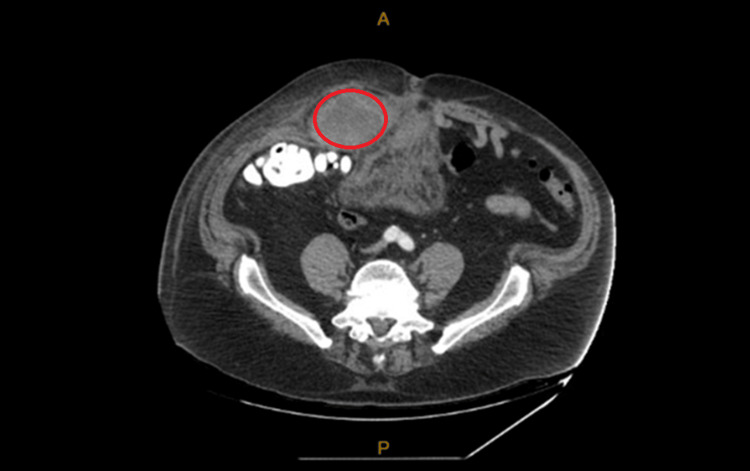
CT of the abdomen with contrast. Red circle showing rectus sheath hematoma measuring 14.6 cm x 8.2 cm.

He was afebrile, with a heart rate of 54 beats per minute, blood pressure of 133/96 mmHg, respiratory rate of 22 breaths per minute, and an oxygen saturation of 99% on room air. He reported mild pain in the right lower quadrant of the abdomen. Initial laboratory tests indicated a hemoglobin level of 13 g/dl, a hematocrit of 37.2%, and a partial thromboplastin time of 84.7 seconds. The prothrombin time was recorded as >90 seconds, and the international normalized ratio (INR) exceeded 8.0 (laboratory value reported on patient chat was INR > 8). The CT scan without contrast ordered as an outpatient was examined and demonstrated a right rectus sheath hematoma measuring 9.4 cm x 6.6 cm x 12.8 cm. A repeat CT of the abdomen and pelvis was done, which showed a rectus sheath hematoma measuring 14.6 cm x 8.2 cm and a small amount of blood extending into the right inguinal canal and tracking inferiorly in the anterior omentum, reaching above the urinary bladder (Figure 2).

The patient received 10 mg of intravenous vitamin K and prothrombin complex concentration. Considering the possibility that interventional radiology might be necessary to manage the suspected bleeding, it was resolved to transfer the patient to our institution for a higher level of care.

Upon arrival at our facility, a subsequent INR measurement revealed a decrease from >8 to 2.3, accompanied by a hemoglobin level of 11.1 g/dL. A follow-up CT scan of the abdomen and pelvis was also requested. The right rectus sheath hematoma appeared to remain stable in size; however, imaging indicated evidence of active contrast extravasation.

The general surgery team was consulted and advised against surgical intervention at that time. The internal medicine team was also brought in, leading to the patient's admission for further evaluation and management. Upon arrival on the ward, the patient was discovered to be experiencing atrial fibrillation with a rapid ventricular response. He was initiated on metoprolol, resulting in significant improvement in his heart rate, and received one unit of packed red blood cells. On the second day of his admission, the patient's hemoglobin levels continued to decline, reaching 9.7 g/dL as indicated by a complete blood count. The interventional radiology team conducted an urgent assessment and performed a visceral angiogram via femoral access. This procedure identified potential arterial irregularities in the midsection of the right inferior epigastric artery, which could indicate either arterial injury or small branching vessels. Due to ongoing concerns regarding bleeding, the interventional radiology team opted to embolize the affected area using a Terumo CX (Terumo Medical Corporation, NJ, USA) detachable coil to achieve hemostasis. In the subsequent days, the patient was closely monitored through routine complete blood counts and other laboratory tests. Although he had previously expressed concerns about affording a direct oral anticoagulant (DOAC), collaboration with the continuity of care and pharmacy team enabled him to be discharged on apixaban.

## Discussion

This case emphasizes several vital aspects concerning the use of warfarin, particularly for patients who are required to persist with this therapy due to financial barriers or clinical requirements, such as the presence of mechanical heart valves. Although warfarin is a potent anticoagulant, it necessitates careful management owing to its narrow therapeutic range and the significant risk of drug interactions that can lead to serious bleeding complications [5,6]. Warfarin is metabolized through the cytochrome P450 (CYP) system, involving enzymes like CYP2C9, CYP1A2, and CYP3A4. Additionally, the effects of warfarin are influenced by polymorphisms in the vitamin K epoxide reductase complex 1 (VKORC1) enzyme [5,6,7]. Consequently, the presence of CYP inhibitors such as amiodarone, isoniazid, and ritonavir can elevate the INR and increase the likelihood of adverse bleeding events in patients taking these medications alongside warfarin due to their effect on the cytochrome P450 system [7]. The multitude of medications that interact with the CYP enzyme system makes it difficult to anticipate which drugs may result in a supratherapeutic INR. These intricacies necessitate ongoing close monitoring and present potential risks with the introduction of any new medications.

The interaction between warfarin and doxycycline in this instance is particularly significant. Doxycycline has the potential to amplify the anticoagulant effects of warfarin by displacing it from its binding sites on serum albumin, and it is believed to inhibit CYP3A4, a component of the cytochrome system involved in warfarin metabolism, which may result in elevated warfarin levels and lead to supratherapeutic outcomes [6,7,8]. It is important to note that doxycycline is not the sole antibiotic that binds to albumin; β-lactam antibiotics and fluoroquinolones also exhibit a primary affinity for albumin. A study conducted by Baillargeon et al. (2002) revealed that long-term use of warfarin in conjunction with any antibiotic was associated with increased INR levels, thereby heightening the risk of bleeding, particularly in individuals aged 65 and older [9]. Furthermore, research by Dowd et al. (2012) demonstrated that a randomized controlled trial involving empiric warfarin dose reduction in patients initiated on doxycycline found that such reductions sometimes resulted in supratherapeutic INR levels; however, routine monitoring of INR levels beginning on the third day of doxycycline administration proved effective in preventing supratherapeutic INR levels through dosage adjustments [10]. Consequently, healthcare professionals should remain vigilant regarding potential drug interactions in patients receiving warfarin. Regular assessments of these patients' medication regimens, including over-the-counter medications and supplements, are crucial. Additionally, dietary considerations are vital, as certain foods, such as green leafy vegetables, avocados, and olive oil, can elevate INR levels, with green leafy vegetables increasing vitamin K levels and diminishing the effectiveness of warfarin.

It is essential to consider several key differential diagnoses in patients exhibiting a supratherapeutic INR, such as liver disease, the presence of lupus anticoagulant, and vitamin K deficiency. In the case at hand, the patient demonstrated normal liver enzyme levels and had no prior history of liver disease. A CT scan of the abdomen ruled out cirrhosis, and lupus anticoagulant levels were found to be within the normal range, indicating that the patient did not meet the diagnostic criteria. This level of vigilance is particularly crucial, as some patients on warfarin who sought medical evaluation for upper respiratory infections have shown INR increases of 5.0 or higher, irrespective of antibiotic treatment. There is a pressing need for further guidelines to enhance the selection of antibiotics for these patients. Moreover, recommendations should clarify whether adjustments to warfarin dosages are necessary and emphasize the importance of more frequent INR monitoring during illness, even in outpatient settings. Finally, healthcare teams should ensure that eligible patients, particularly those facing financial difficulties, have access to safer alternative direct oral anticoagulants (DOACs), as demonstrated in this case through patient assistance programs or insurance navigation support.

## Conclusions

We reported a case involving a significant rectus sheath hematoma accompanied by active bleeding, which arose following a treatment regimen of doxycycline in a patient receiving warfarin. Although there are documented instances of drug interactions with warfarin that can lead to hematoma formation, occurrences of this particular interaction with doxycycline resulting in a substantial, life-threatening hematoma are rarely reported. This situation emphasizes the necessity for healthcare professionals to remain alert to potential drug interactions, particularly in patients undergoing warfarin anticoagulation therapy. Clinicians should explore alternative antibiotics with examples such as azithromycin, Augmentin, and others to lessen the likelihood of such adverse outcomes. Furthermore, this case highlights the need for ongoing patient education regarding the signs and symptoms of bleeding complications associated with anticoagulation therapy. Despite the decreasing use of warfarin, increased awareness and proactive management can significantly mitigate the risks linked to drug interactions.
